# Maladaptive Self- and Interpersonal Functioning Increments General Psychiatric Severity in the Association with Adolescent Personality Pathology

**DOI:** 10.3390/children10010120

**Published:** 2023-01-06

**Authors:** Carla Sharp, Breana Rachelle Cervantes

**Affiliations:** Department of Psychology, University of Houston, 126 Heyne Building, Houston, TX 77004, USA

**Keywords:** ICD-11, alternative model for personality disorders, adolescents, level of personality functioning

## Abstract

Dimensionalized diagnostic systems, especially the entry criterion of maladaptive self and interpersonal functioning, hold particular advantages for the downward extension of personality pathology to young persons, but require conceptual clarification. The current study evaluated the distinctiveness of maladaptive self and interpersonal functioning by examining its incremental value over and above general psychiatric severity in the association with personality pathology. A community sample of *N* = 419 youth (50.4% female; *M_age_* = 11.91, *SD* = 1.19) between the ages of 10 and 14 completed measures of maladaptive self- and interpersonal functioning, general psychiatric severity (internalizing–externalizing spectrum), and personality pathology. Results showed that, as expected, maladaptive self- and interpersonal functioning incremented general psychiatric severity in the association with personality pathology in adolescents. Results contribute to the literature base illustrating the value of the entry criterion of the ICD-11 and AMPD diagnostic system.

## 1. Introduction

It is now widely recognized that dimensional models of psychopathology, including that of personality pathology, hold several advantages for more accurate assessment and diagnosis, thereby increasing the likelihood of personalized care [1,2]. Accordingly, the 11th version of the International Classification of Diseases (ICD-11) adopted a radically new conceptualization of personality disorder, which took effect at the beginning of 2022 [3]. The recent ICD-11 innovation was foreshadowed by inclusion of a dimensionalized model of personality disorder in Section III of the 5th edition of the Diagnostic and Statistical Manual of Mental Disorders (DSM-5 [4])—the so-called Alternative Model for Personality Disorders (AMPD). The new ICD-11 model for personality disorder is viewed as basically identical to the AMPD [5], although the ICD-11 system may be regarded as perhaps more streamlined, with several features reducing complexity (e.g., making the use of trait domains optional, removing all trait facets, and removing the option of hybrid categories of personality disorders).

Instead of following the tradition of the ICD-10 and DSM-IV-TR of 10 polythetic, categorical disorders, the ICD-11 and the AMPD systems recommend that the clinician first evaluate general (global) impairment in self- and interpersonal functioning. The entry criterion for personality pathology, maladaptive self- and interpersonal functioning is viewed as the core and common feature of all personality pathology, regardless of its specific flavor. In the ICD-11, maladaptive self- and interpersonal functioning is classified according to overall severity (Mild, Moderate, Severe) or may be classified as sub-threshold (i.e., Personality difficulty). This system roughly corresponds to the Levels of Personality Functioning (LPF) in the AMPD which is rated from 0 (typical personality functioning) to 4 (severely maladaptive personality functioning). Next, the ICD-11 gives the clinician the option to specify one or more maladaptive trait domains (negative affectivity, detachment, dissociality, disinhibition and anankastia). These five trait domains correspond to the AMPD’s maladaptive trait domains with the exception of anankastia, which in the AMPD is viewed as the opposite extreme of disinhibition, with psychoticism added as the fifth maladaptive trait domain. In a third (optional) step, the clinician can then specify whether the pattern of problems corresponds to a borderline pattern (ICD-11) or borderline, schizotypal, antisocial, narcissistic, obsessive–compulsive, or avoidant pattern (AMPD). This last step is seen by many in the field as redundant as these categories are well represented in either the general severity criterion, in the case of borderline personality disorder (BPD) [6], or the maladaptive trait domains [7,8,9,10,11,12].

Strong support, both conceptually and empirically, has been published for both the AMPD [13,14,15,16] and ICD-11 [5,17,18] systems. A particular advantage of dimensionalized diagnostic systems lies in the benefit they hold for downward extension to children and adolescents [19,20,21]. Psychopathology in all individuals, and more so in children and adolescents, is known to wax and wane at the phenotypic level, even when environmental and/or neurobiological and genetic risk markers stay quite constant throughout development. As such, a dimensionalized diagnostic system allows the tracking of phenotypic manifestations of underlying psychopathology over time, acknowledging that sub-threshold expression may be clinically as meaningful as manifestations above the clinical threshold. Importantly, dimensionalized diagnostic systems also allow for the development of clinical staging approaches to the assessment and treatment of psychopathology. Indeed, AMPD-informed clinical staging assessment [22] and treatment [23,24,25] protocols for personality pathology in youth have been proposed.

Of particular developmental relevance is the ICD-11 and AMPD entry criterion of maladaptive self- and interpersonal functioning. This is because the onset of personality disorder coincides with qualitative developmental shifts in these domains [26,27,28,29]. Developmental research suggests that while individual differences in dispositional traits are readily observable in children as young as infancy, extreme traits in and of themselves are not necessarily indicative of personality disturbance. It is not until adolescence, when adult-like features in self- and interpersonal functioning begin to emerge that personality disturbance becomes detectable. Specifically, the adolescent’s ability to make sense of their identity in the context of increasingly complex and changing relationships with parents, peers, and romantic others, and to manage and regulate themselves in this context, becomes the yardstick by which healthy personality development is measured. Put differently, maladaptive traits may represent the continuous, descriptive aspects of personality, while the management of self in the context of interpersonal relationships expresses the process-oriented aspects of personality that begin to bind together in adolescence [30]. This approach to understanding personality and personality disorder is not only developmentally rigorous, but also aids in destigmatization of a highly stigmatized condition. In making the assessment of maladaptive traits optional (and not conditional for the diagnosis of personality disorder), it de-emphasizes the dispositional aspects of personality, and instead brings into prime focus a malleable treatment target—that is, the improvement of the management and regulation of the self in the context of interpersonal relationships. In other words, disorder lies not in who you are as a person, but how you manage yourself in the context of relationships.

However, for this aspect of personality functioning to reach its full potential as a developmental marker of personality pathology, it is important that the entry criterion for personality disorder in the ICD-11 and AMPD be clearly defined. Weaknesses reflecting potential confusion over its definition have been highlighted. For instance, it has been noted that the ICD-11 global severity determination, without considering the traits (which are optional in the ICD-11 system), may be “vague, imprecise and therefore not very informative”, and that the global severity classification may be “too minimalistic and unsophisticated for specialist clinical practice” ([17], p. 4). Concerns over potential vagueness, imprecision, and minimalist nature of the entry criterion seem to be based on confusion of the current entry criterion with the initial ICD-11 conceptualization of general personality functioning as nothing more than severity in general dysfunction. This view was originally proposed by Tyrer [31] who suggested that severity of personality dysfunction is best viewed as the impact of extreme traits on interpersonal functioning. In this view, general personality functioning is the consequence of maladaptive traits and not its source. Such a view stands in contrast with the purpose of the LPF intended by the DSM-5 workgroup as a meaning-making system that drives trait function [32,33] and was therefore rectified during the ISSPD meeting of 2018 in Heidelberg when maladaptive self function was added back into the ICD-11 entry criterion [34]. This, alongside interpersonal dysfunction, forms the core and common feature that distinguishes personality dysfunction from other forms of psychopathology. Despite this adjustment, disagreement lingers over the exact meaning of the entry criterion, resulting in arguments that general personality functioning is well represented by the general psychiatric severity, which in factor analytic studies is referred to as the p-factor [35], or in other approaches simply denotes the total sum of psychopathology [36], or high severity in the neuroticism or negative affectivity as representative of the p-factor [37], or an interaction of maladaptive traits resulting in maladaptive interpersonal signatures [38]. Regardless of the specific argument, they all have in common a view that maladaptive traits alone are sufficient to describe (and diagnose) personality disorder rendering the entry criterion of maladaptive self- and interpersonal functioning redundant [39].

Against this background, studies have begun to examine the incremental value especially of the self-components of the entry criterion in order to show that it is not redundant. Most studies in this regard have focused on distinguishing maladaptive self function from general psychosocial disability in adults [40], and more recently, adolescents [41]. Fewer studies, thus far, have aimed at showing that maladaptive self-and interpersonal functioning is distinguished from general psychiatric severity in explaining personality difficulties. To this end, the current study aimed at evaluating the incremental utility of maladaptive self and identity function, as well as LPF, over that of general psychiatric severity (i.e., total sum of internalizing and externalizing psychopathology) in predicting PD-relevant outcomes in adolescence. Based on the theoretical position outlined above, we expected measures of maladaptive self- and identity function and/or measures of LPF to make an incremental contribution in predicting individual differences in personality pathology over and above general psychiatric severity in a community sample of young adolescents.

## 2. Materials and Methods

### 2.1. Participants and Procedure

The sample consisted of a total of *N* = 419 youth (50.4% female; *M_age_* = 11.91, *SD* = 1.18) between the ages of 10 and 14 who attended a public charter middle school for grades 5 to 8 in a large, metropolitan city in the southwestern United States. Study inclusion criteria include being an adolescent between ages 10–18, attending regular school, and English proficiency. Exclusion criteria were being a child younger than 10 years or an adult older than 18 years. Notably, the school is situated in the most densely populated neighborhood of the city, which is a predominantly low-income, Latinx/Hispanic immigrant community. Thus, the demographics of the student population are reflective of the surrounding neighborhood. The sample was characterized by the following racial and ethnic composition: 70.7% Hispanic, 9.1% Black/African American, 5.8% Asian, 5.3% multiple races, 1.9% White, 1.0% American Indian, and 6.2% “other” race.

The present study was approved by the appropriate human subjects review committee. As part of a larger study, all students attending the school were informed of a research opportunity to complete an assessment during school. Parental consent was waived, as questionnaires were anonymous and did not pose more than minimal risk. Positive informed assent was obtained from interested students, and surveys were subsequently administered using Qualtrics survey software during a single 45–50-min class period. Only data obtained from the baseline assessment were examined for the present study. Although response rates vary for the select measures used in the present study, these differences are random for most measures, except for the PID-5-BF. With respect to the PID-5-BF, the smaller number is likely explained by the fact that students were allotted a limited amount of time to complete the questionnaires and some students may not have reached the PID-5-BF, which was programmed at the end of the survey battery. Indeed, the sample of participants who completed the PID-5-BF (*n* = 269) versus those who did not (*n* = 150) significantly differs in terms of age (see Results), which suggests that a higher number of older students were able to complete the PID-5-BF in the allotted time than younger students. Table 1 summarizes sample demographics.

### 2.2. Measures

#### 2.2.1. Evaluation of the Entry Criterion for Personality Disorder

Maladaptive self- and interpersonal functioning was evaluated in two ways. First, we used the Levels of Personality Functioning 12-18 (LoPF-Q 12-18 [42]). The LoPF-Q 12-18 is a 97-item self-report measure of impairment in personality functioning, as described by Criterion A of the DSM-5 Section III AMPD, that yields a total score of personality disorder severity and four subscales corresponding to the four dimensions of Criterion A (Identity, Self-direction, Empathy, and Intimacy). Participants rate items on a 5-point Likert-scale ranging from 0 (*no*) to 4 (*yes*). In the present study, the English translation was administered [43]. Only the total score was examined in the present study. The LoPF-Q 12-18 has demonstrated good to excellent psychometric properties in previous studies of German and Turkish adolescents [44,45]. Although initially developed for use with youth ages 12 to 18, the LoPF-Q 12-18 has been validated for 11–20-year-olds in a sample of inpatient and outpatient German youth [44] and 10–18-year-olds in an American community sample [46]. Of the total 419 study participants, *n* = 404 completed this measure. In the current study, internal consistency was high (α = 0.94).

As an additional measure specifically of the self-focused aspects of the entry criterion, we used the Assessment of Identity Development in Adolescence (AIDA [47]), which is a 58-item questionnaire asking respondents to rate items on a 5-point Likert-type scale, ranging from 0 (*I strongly disagree*) to 4 (*I strongly agree*). In the present study, the American culture-adapted translation of the AIDA was administered [48]. The total maladaptive identity score, representing identity diffusion, is comprised of two subscales (Discontinuity and Incoherence). The total identity diffusion score was evaluated in the present study. Reliability and validity of the AIDA have been established in several samples of culturally diverse adolescents e.g., [49,50,51]. In the subsample of participants that completed the AIDA (*n* = 415), Cronbach’s alpha indicated high internal consistency (α = 0.92).

#### 2.2.2. Evaluation of General Psychiatric Severity

To assess general psychiatric severity, we used the Brief Problem Monitor (BPM [52]) which provides an index of internalizing-externalizing spectrum disorder severity. Informed by the Achenbach System of Empirically Based Assessment (ASEBA [53]) instruments, the BPM is a 19-item questionnaire that yields 4 subscales (Total Problems, Internalizing Problems, Externalizing Problems, Attention Problems). Responses are rated on a 3-point Likert-type scale ranging from 0 to 2 (0 = *not true*, 1 = *somewhat true*, 2 = *very true*) with higher scores indicating greater severity of emotional and behavioral problems. For the present study, total raw scores were calculated to obtain the overall level of psychopathology symptom severity (BPM Total Problems). The BPM has previously demonstrated adequate psychometric properties in a large community sample of youth [52,54]. Cronbach’s alpha was 0.89 in the present study. A total of *n* = 381 participants completed this measure.

#### 2.2.3. Individual Differences in Personality Pathology

To evaluate the dependent variable (individual differences in personality pathology) we included first the Personality Inventory for DSM-5 Brief Form (PID-5-BF [55]). The PID-5-BF is a reduced, 25-item self-report measure derived from the original 220-item PID-5 [56]. The PID-5-BF assesses pathological personality traits, as described by Criterion B of the DSM-5 Section III AMPD. Each item is rated on a 4-point Likert-type scale (0 = *very false or often false*, 1 = *sometimes or somewhat false*, 2 = *sometimes or somewhat true*, 3 = *very true or often true*). Items are summed to compute a total score of maladaptive personality traits and five domain scores (Negative Affect, Detachment, Antagonism, Disinhibition, and Psychoticism). Higher scores indicate greater personality dysfunction. The PID-5-BF has previously been validated in samples of Italian and Spanish youth [57,58], and only 1 sample of American youth [59]. In the present study, PID-5-BF data were obtained from *n* = 269 participants. Cronbach’s alpha indicated high internal consistency (α = 0.92).

As a second index of individual differences in personality pathology, we administered the Borderline Personality Features Scale for Children-11 (BPFSC-11 [60]), which is an 11-item self-report measure of borderline features among children and adolescents originally derived from the full 24-item BPFSC [61]. Items are rated on a 5-point Likert scale (1 = *not at all true*, 2 = *hardly ever true*, 3 = *sometimes true*, 4 = *often true*, 5 = *always true*) and summed to calculate a total score and four subscales (Affective Instability, Identity Problems, Negative Relationships, Self-harm). Higher scores indicate greater levels of borderline personality symptoms. The total sum score was used for the current study. The BPFSC-11 has demonstrated adequate psychometric properties in previous studies [60] In the current study, *n* = 406 participants completed the BPFSC-11. The BPFSC-11 demonstrated good internal consistency (α = 0.82).

### 2.3. Data Analytic Strategy

#### 2.3.1. Attrition Analyses

Analyses were conducted in SPSS Version 29 [62]. Attrition analyses were conducted to examine whether there were any systematic differences in age, gender, maladaptive identity (AIDA), maladaptive self- and interpersonal functioning (LoPF), total internalizing-externalizing problems (BPM), and borderline traits (BPFSC-11) between adolescents that completed the PID-5-BF and those that did not. Comparisons were made using independent samples *t*-tests and chi-square analyses.

#### 2.3.2. Descriptive Statistics

Descriptive statistics were computed to examine bivariate relations between study variables: maladaptive identity (AIDA), maladaptive self and interpersonal (LoPF), general psychiatric severity (BPM total problems), maladaptive personality traits (PID-5-BF) and borderline features (BPFSC-11), and age and gender were also examined as possible covariates. Gender differences in all study variables were tested using *t*-tests. Additionally, relations between continuous variables were examined using Pearson’s correlations.

#### 2.3.3. Regression Analyses

To examine the incremental utility of LoPF maladaptive self- and interpersonal functioning in predicting personality pathology over and above age, gender, and overall emotional and behavioral problems, we conducted two hierarchical linear regressions. In the first model, age and gender were entered at Step 1, BPM total problems was entered at Step 2, and LoPF was entered at Step 3. The dependent variable was the PID-5-BF. In the second model, we conducted a similar linear regression analysis with BPFSC-11 entered as the dependent variable. In Step 1, we entered age and gender. BPM total problems was entered at Step 2, and LoPF was entered at Step 3.

Next, to examine the incremental utility of AIDA maladaptive identity in predicting personality pathology beyond age, gender, and overall emotional and behavioral problems, we conducted another series of hierarchical linear regression analyses. In the third model, age and gender were entered at Step 1, BPM total problems was entered at Step 2, and AIDA was entered at Step 3. The dependent variable was the PID-5-BF. In the fourth model, BPFSC-11 was entered as the dependent variable. Age and gender were entered at Step 1, BPM total problems at Step 2, and AIDA at Step 3.

For all models, multicollinearity was estimated using tolerance and the variance inflation factor. Because of the significant bivariate relations between gender and scores on BPM total problems, BPFSC-11, AIDA, and LoPF, gender was included as a covariate in all four regression models. Age was also included as a covariate, although only marginally associated with AIDA at the bivariate level.

## 3. Results

### 3.1. Attrition Analyses

We conducted independent sample *t*-tests for continuous variables (age, AIDA, LoPF, BPM, BPFSC-11) and a chi-square test for the categorical variable (gender) between PID-5-BF completers and non-completers. Results showed no significant differences between PID-5-BF completers and non-completers in AIDA maladaptive identity (*t*(413) = –1.80, *p* = 0.072) or gender (χ2 (2, *N* = 419) = 1.01, *p* = 0.602). However, significant differences were found in age (*t*(293.75) = -3.94, *p* < 0.001), with PID-5-BF completers (*M* = 12.08, *SD* = 1.11) being significantly older than non-completers (*M* = 11.61, *SD* = 1.25). Further, significant differences were also found for LoPF maladaptive self- and interpersonal functioning (*t*(229.22) = −8.93, *p* < 0.001), BPM total problems (*t*(379) = −3.73, *p* < 0.001), and BPFSC-11 borderline features (*t*(308.77) = −2.01, *p* = 0.046). Completers had significantly higher LoPF scores (*M* = 188.22, *SD* = 50.79) than non-completers (*M* = 129.92, *SD* = 68.45); significantly higher BPM total problems (*M* = 14.18, *SD* = 7.70) than non-completers (*M* = 11.14, *SD* = 6.99); and significantly higher BPFSC-11 borderline traits (*M* = 31.23, *SD* = 8.72) than non-completers (*M* = 29.49, *SD* = 8.16).

### 3.2. Descriptive Statistics

Table 2 provides descriptive statistics and Pearson’s correlations among main study variables.

All main study variables demonstrated significant relations with one another. Of particular interest to the present study, higher levels of LoPF were strongly associated with higher levels of BPM total problems, BPFSC-11, and PID-5-BF. In addition, AIDA scores were highly correlated with BPM total problems, BPFSC-11, and PID-5-BF. A weak, negative correlation between age and AIDA score was demonstrated, such that younger age was associated with higher levels of identity diffusion. Results from *t*-tests revealed that females had significantly higher scores than males on LoPF (*M* = 178.29 vs. 154.72, *t*(398) = 3.76, *p* < 0.001), AIDA (*M* = 118.90 vs. 107.23, *t*(409) = 3.64, *p* < 0.001), BPM total problems (*M* = 13.95 vs. 12.17, *t*(375) = 2.31, *p* < 0.02), and BPFSC-11 (*M* = 32.29 vs. 28.71, *t*(400) = 4.30, *p* < 0.001). No significant gender differences were demonstrated for age or PID-5-BF maladaptive personality traits.

### 3.3. Regression Analyses

Tolerance (0.51–1.00) and variance inflation factor (1.00–1.97) were within acceptable limits in all regression models. Table 3 provides results of the regression models examining the added value of the LoPF in predicting personality pathology.

In the first series of regressions, we examined the added value of LoPF maladaptive self- and interpersonal functioning over and above age, gender, and BPM total problems in predicting PID-5-BF maladaptive personality traits. In Step 1, the overall model was nonsignificant, *F*(2, 256) = 0.892, *p* = 0.411. Age and gender were not significantly associated with PID-5-BF scores. In Step 2, the overall model was significant, *F*(3, 255) = 90.431, *p* < 0.001. Only BPM total problems score was a significant predictor of PID-5-BF maladaptive personality traits. In Step 3, when the LoPF variable was added to the model, the overall model retained significance, *F*(4, 254) = 89.185, *p* < 0.001. Both BPM total problems and LoPF exhibited significant relations with the PID-5-BF. Adjusted R^2^ increased from 51.0% to 57.8%, reflecting a 6.8% change in the explained variance of the PID-5-BF scores with the addition of LoPF total personality disorder severity to the model.

The next series of regressions was conducted to test the predictive utility of LoPF maladaptive self- and interpersonal functioning in estimating BPFSC-11 borderline features, beyond age, gender, and BPM total problems. In Step 1, the overall model was significant, *F*(2, 377) = 8.246, *p* < 0.001. Age was not significantly associated with borderline features. Gender demonstrated significant relations with BPFSC-11 scores. In Step 2, the overall model retained significance (*F*(3, 376) = 165.506, *p* < 0.001), with the addition of the BPM total problems variable. Both gender and BPM total problems were significantly associated with scores on the BPFSC-11. In Step 3, the overall model continued to be significant, *F*(4, 375) = 133.812, *p* < 0.001. Gender, BPM total problems, and LoPF were significant predictors of borderline features. When the LoPF variable was added to the model, adjusted R^2^ increased from 56.6% to 58.4%, resulting in a 1.8% change in adjusted R^2^ values.

Next, we examined the incremental validity of AIDA maladaptive identity in predicting PID-5-BF maladaptive personality traits over and above age, gender, and BPM total problems. Step 1 and Step 2 were identical to those computed in the model assessing the incremental validity of LoPF in predicting PID-5-BF traits. Thus, in Step 1, neither age nor gender exhibited significant relations with PID-5-BF scores, and in Step 2, only BPM total problems was significantly associated with the PID-5-BF. In Step 3, the overall model continued to be significant, *F*(4, 254) = 73.143, *p* < 0.001. Both BPM total problems and AIDA demonstrated significant associations with PID-5-BF scores. The change in adjusted R^2^ values was 1.8% (from 51% to 52.8%), indicating a 1.8% change in the explained variance of the PID-5-BF scores due to the addition of AIDA maladaptive identity to the model.

In the final set of regressions, we tested the added value of AIDA maladaptive identity beyond age, gender, and BPM total problems in predicting BPFSC-11 borderline features. We computed Step 1 and Step 2 exactly as we had done in the model assessing the incremental validity of AIDA in predicting BPFSC-11 borderline features. Accordingly, in Step 1, gender, but not age, was significantly associated with borderline features, and in Step 2, both gender and BPM total problems were related to borderline features. In Step 3, the overall model retained significance, *F*(4, 375) = 183.250, *p* < 0.001. Gender, BPM total problems, and AIDA each demonstrated significant relations with BPFSC-11 scores. Adjusted R^2^ increased from 56.6% to 65.8%, indicating a 9.2% change in the explained variance in the BPFSC-11 scores due to the addition of the AIDA variable to the model.

The results of the two models assessing the incremental validity of the AIDA in predicting dimensions of personality pathology are summarized in Table 4.

## 4. Discussion

The current study aimed to evaluate whether measures of the construct represented by the entry criterion of the ICD-11 and AMPD (maladaptive self- and/or interpersonal functioning) provide added value over and above a measure of general psychiatric severity in association with personality pathology. Our results showed that both maladaptive self- and interpersonal functioning (the LoPF) and maladaptive self- and identity functioning (AIDA) incremented general psychiatric severity when personality pathology was measured by either a maladaptive trait measure (PID-5-BF) or a measure of borderline traits (BPFSC-11). The incremental value was most pronounced for the AIDA and the BPFSC-11 (9.2%) and the LoPF and the PID-5-BF (6.8%), albeit statistically significant also for the other combinations of independent and dependent variables. It is worth noting that despite moderately high correlations with general psychiatric severity (i.e., 0.65 for the AIDA and 0.53 for the LoPF), measures representing the entry criterion of the ICD-11 and/or AMPD, still incremented psychiatric severity in its association with personality pathology; and that incremental value held even when the sample size was reduced when the PID-5 was the dependent variable. We also note that the correlation between general psychiatric severity and maladaptive traits was higher (0.72) than that of the AIDA and LoPF, suggesting greater overlap in constructs measured by the BPM and PID-5-BF. This fits with the personality–psychopathology spectrum approach that underlies new quantitative approaches to reconceptualizing psychopathology like HiTOP [1], in which maladaptive trait function is well represented by the internalizing–externalizing dimensions of psychopathology. Our own view has been that these dimensions are not quite enough to capture personality pathology and the current results make a small contribution in confirming this. Put differently, if the internalizing–externalizing spectra were adequate in covering personality pathology, measures of maladaptive self- and/or interpersonal functioning would not have incremented the BPM in its association with total borderline score or total maladaptive trait score.

Taken together, our results support the position that maladaptive self- and interpersonal functioning should not be equated with general psychiatric severity. These findings are consistent with the results from studies that have investigated whether LPF can be distinguished from general psychosocial disability in adults [40,63] and adolescents [41]. While general psychosocial disability is a different construct from general psychiatric severity, the fact that maladaptive self- and interpersonal functioning seems to distinguish itself from these measures in its association with measures of personality pathology provides evidence in support of the importance of the entry criterion for diagnosing personality pathology through incremental validity analyses. This question can also be approached from a factor-analytic point of view which may result in different conclusions. Using other personality-disorder relevant outcomes may also result in different conclusions. For instance, maladaptive self- and interpersonal functioning may be of particular relevance for borderline pathology and maladaptive trait function as measured here but may be less predictive of other personality-disorder relevant outcomes.

Despite these limitations, the fact that maladaptive self- and interpersonal functioning demonstrated incremental value over and above general psychiatric severity in adolescents, potentially addresses concerns over the developmental implications for the reconceptualization of personality disorder in dimensional terms as now legitimized in the ICD-11. Concerns may include pathologizing youth personality functioning when the clinical threshold is not met; or pathologizing general functioning that may be a consequence of adverse living circumstances rather than personality pathology per se. The results of the current study are of particular relevance for these concerns because our results emphasize the importance of not viewing the entry criterion of the ICD-11 as general impairment, general disability, general functioning, or general psychiatric severity [64]. If the ICD-11 entry criterion is viewed thus, we share the concerns over pathologizing youth unnecessarily. However, if the ICD-11 criterion is viewed as the process-orientated meaning-making aspects of personality functioning, then it is not a mere consequence of extreme levels in maladaptive traits (or adversity), but the source of personality difficulties. Thus, rather than viewing the entry criterion as merely descriptive of level of general functioning, the entry criterion (to be non-pathologizing) must denote how impaired an individual is in making sense of their experiences in a coherent way to facilitate an integrated sense of self. In personality disorder, it is this intrapsychic meaning-making system, subserved by the capacity to mentalize self and others, that has broken down. We can therefore imagine two individuals who display a level of intensity in dispositional traits to the same degree (e.g., emotional reactivity, negative affectivity, and disinhibition) both living in adverse circumstances where these traits are further exacerbated. One individual will slow down to make sense of their dispositions in the context of their circumstances, integrate them into a sense of self, and negotiate them to fit with the context. The other will be overwhelmed, frustrated, and confused by their dispositional traits leading to an unwillingness or incapacity to integrate them into a coherent sense of self, resulting in failure to navigate and adapt to context demands. This process of meaning-making is what constitutes the core of personality disorder—not the severity of the traits or general function, but something that happens in the mind when individuals try to make sense of their subjective experience. If conceptualized thus, we view the reconceptualization of personality disorder in the ICD-11 as a significant advance, because it provides a clinically significant and developmentally relevant entry criterion that captures the process that goes awry specifically in personality pathology. It also allows the acknowledgement that this capacity ebbs and flows and that a young person below clinical threshold may still need scaffolding to make sense of who they are to successfully negotiation context demands. We also view such reconceptualization as destigmatizing because it denotes the desperate attempt of a human being to make sense of themselves and their experiences rather than a descriptive account of who they are as a person.

## 5. Conclusions

This paper contributes to the argument supporting the dimensionalization of personality pathology for its advantages for developmental perspectives on personality functioning. It reiterates previous conclusions of the importance of maladaptive self- and interpersonal functioning as conditional to the diagnosis of personality disorder—a thesis legitimized in the recent ICD-11 conceptualization of personality pathology.

## Figures and Tables

**Table 1:** Sample demographics.

Variable	*N* = 419 (%)
Gender	
Female	50.4
Male	48.7
Other	1.0
Ethnicity ^1^	
Hispanic/Latinx	70.7
Black/African American	9.1
Asian	5.8
White	1.9
American Indian	1.0
Multiple races	5.3
Other	6.2
Grade	
Fifth	27.2
Sixth	27.7
Seventh	23.9
Eighth	21.2
Age, mean (SD) ^1^	11.91 (1.18)

^1^ Missing data from *n* = 2 for ethnicity and *n* = 1 for age.

**Table 2:** Bivariate relations among study variables.

Variable	1.	2.	3.	4.	5.	6.
1. LoPF Maladaptive self and interpersonal (*n* = 404)						
2. AIDA Maladaptive identity (*n* = 415)	0.61 **					
3. BPM General psychiatric severity (total problems) (*n* = 381)	0.53 **	0.65 **				
4. PID-5-BF Maladaptive personality traits (*n* = 269)	0.65 **	0.59 **	0.72 **			
5. BPFSC-11 Borderline features (*n* = 406)	0.52 **	0.74 **	0.74 **	0.72 **		
6. Age	0.04	−0.11 *	0.08	−0.10	0.02	
Mean	167.58	113.30	13.18	1.14	30.62	11.91
SD	63.99	32.90	7.60	0.58	8.56	1.18
Skew	−0.55	−0.50	0.31	0.03	−0.16	−0.002
Kurtosis	−0.29	0.86	−0.46	−0.05	−0.30	−0.91

Note. LoPF = Levels of Personality Functioning; AIDA = Assessment of Identity Development in Adolescence; BPM = BPM = Brief Problem Monitor; PID-5-BF = Personality Inventory for DSM-5 Brief Form; BPFSC-11 = Borderline Personality Features Scale for Children—11 items. * *p* < 0.05; ** *p* < 0.001.

**Table 3:** Hierarchical regression models testing the incremental validity of LoPF maladaptive self- and interpersonal functioning.

Variable		b	*SE*	β	*t*	*p*	Tol.	VIF	Adj. R^2^	Δ Adj. R^2^
*DV* = Maladaptive Personality Traits (PID-5-BF Total Score)										
Step 1 ^a^	Age	−0.04	0.03	−0.07	−1.08	0.28	1.00	1.00	−0.1%	
	Gender	−0.05	0.07	−0.05	−0.71	0.48	1.00	1.00		
Step 2 ^b^	Age	−0.03	0.02	−0.06	−1.41	0.16	1.00	1.00	51.0%	51.1%
	Gender	0.02	0.05	0.02	0.37	0.71	0.99	1.01		
	BPM Total Problems	0.05	0.003	0.72	16.36	<0.001	0.99	1.01		
Step 3 ^c^	Age	−0.03	0.02	−0.05	−1.33	0.18	1.00	1.01	57.8%	6.8%
	Gender	0.04	0.05	0.04	0.93	0.35	0.98	1.02		
	BPM Total Problems	0.04	0.004	0.50	9.56	<0.001	0.60	1.68		
	LoPF Maladaptive Self and Interpersonal	0.004	0.001	0.34	6.47	<0.001	0.59	1.69		
*DV* = Borderline Personality Features (BPFSC-11 Total Score)										
Step 1 ^d^	Age	0.45	0.37	0.06	1.21	0.23	0.99	1.01	3.7%	
	Gender	−3.24	0.82	−0.20	−3.97	<0.001	0.99	1.01		
Step 2 ^e^	Age	−0.003	0.25	<0.001	−0.013	0.99	0.99	1.02	56.6%	52.9%
	Gender	−2.16	0.55	−0.13	−3.93	<0.001	0.98	1.02		
	BPM Total Problems	0.81	0.04	0.73	21.45	<0.001	0.99	1.01		
Step 3 ^f^	Age	0.01	0.24	0.001	0.03	0.98	0.99	1.02	58.4%	1.8%
	Gender	−1.97	0.54	−0.12	−3.64	<0.001	0.98	1.02		
	BPM Total Problems	0.72	0.04	0.65	16.56	<0.001	0.72	1.39		
	LoPF Maladaptive Self and Interpersonal	0.02	0.01	0.16	4.15	<0.001	0.72	1.39		

Note. PID-5-BF = Personality Inventory for DSM-5 Brief Form; BPM = Brief Problem Monitor; LoPF = Levels of Personality Functioning; BPFSC-11 = Borderline Features Scale for Children—11 items. Gender coded as a dichotomous variable: 0 = female, 1 = male. ^a^ model nonsignificant, *F*(2, 256) = 0.892, *p* = 0.411; ^b^ model significant, *F*(3, 255) = 90.431, *p* < 0.001; ^c^ model significant, *F*(4, 254) = 89.185, *p* < 0.001. ^d^ model significant, *F*(2, 377) = 8.246, *p* < 0.001; ^e^ model significant, *F*(3, 376) = 165.506, *p* < 0.001; ^f^ model significant, *F*(4, 375) = 133.812, *p* < 0.001.

**Table 4:** Hierarchical regression models testing the incremental validity of AIDA maladaptive identity.

Variable		b	*SE*	β	*t*	*p*	Tol.	VIF	Adj. R^2^	Δ Adj. R^2^
*DV* = Maladaptive Personality Traits (PID-5-BF Total Score)										
Step 1 ^a^	Age	−0.04	0.03	−0.07	−1.08	0.28	1.00	1.00	−0.1%	
	Gender	−0.05	0.07	−0.05	−0.71	0.48	1.00	1.00		
Step 2 ^b^	Age	−0.03	0.02	−0.06	−1.41	0.16	1.00	1.00	51.0%	51.1%
	Gender	0.02	0.05	0.02	0.37	0.71	0.99	1.01		
	BPM Total Problems	0.05	0.003	0.72	16.36	<0.001	0.99	1.01		
Step 3 ^c^	Age	−0.02	0.02	−0.05	−1.04	0.30	0.98	1.02	52.8%	1.8%
	Gender	0.04	0.05	0.04	0.90	0.37	0.96	1.04		
	BPM Total Problems	0.04	0.004	0.58	9.85	<0.001	0.52	1.91		
	AIDA Maladaptive Identity	0.004	0.001	0.20	3.29	0.001	0.51	1.97		
*DV* = Borderline Personality Features (BPFSC-11 Total Score)										
Step 1 ^d^	Age	0.45	0.37	0.06	1.21	0.23	0.99	1.01	3.7%	
	Gender	−3.24	0.82	−0.20	−3.97	<0.001	0.99	1.01		
Step 2 ^e^	Age	−0.003	0.25	<0.001	−0.01	0.99	0.99	1.02	56.6%	52.9%
	Gender	−2.16	0.55	−0.13	−3.93	<0.001	0.98	1.02		
	BPM Total Problems	0.81	0.04	0.73	21.45	<0.001	0.99	1.01		
Step 3 ^f^	Age	0.34	0.22	0.05	1.51	0.13	0.96	1.04	65.8%	9.2%
	Gender	−1.48	0.49	−0.09	−3.01	0.003	0.97	1.04		
	BPM Total Problems	0.51	0.04	0.47	11.59	<0.001	0.56	1.78		
	AIDA Maladaptive Identity	0.11	0.01	0.41	10.12	<0.001	0.55	1.81		

Note. PID-5-BF = Personality Inventory for DSM-5 Brief Form; BPM = Brief Problem Monitor; AIDA = Assessment of Identity Development in Adolescence; BPFSC-11 = Borderline Features Scale for Children—11 items. Gender coded as a dichotomous variable: 0 = female, 1 = male. ^a^ model nonsignificant, *F*(2, 256) = 0.892, *p* = 0.411; ^b^ model significant, *F*(3, 255) = 90.431, *p* < 0.001; ^c^ model significant, *F*(4, 254) = 73.143, *p* < 0.001. ^d^ model significant, *F*(2, 377) = 8.246, *p* < 0.001; ^e^ model significant, *F*(3, 376) = 165.506, *p* < 0.001; ^f^ model significant, *F*(4, 375) = 183.250, *p* < 0.001.

## Data Availability

De-identified data is available from the first author.

## References

[1] Kotov R., Krueger F.R., Watson D., Cicero C.D., Conway C.C., DeYoung G.C., Eaton N.R., Forbes M.K., Hallquist M.N., Latzman R.D. (2021). The Hierarchical Taxonomy of Psychopathology (HiTOP): A quantitative nosology based on consensus of evidence. Annu. Rev. Clin. Psychol..

[2] Reed G.M., First M.B., Kogan C.S., Hyman S.E., Gureje O., Gaebel W., Maj M., Stein D.J., Maercker A., Tyrer P. (2019). Innovations and changes in the ICD-11 classification of mental, behavioural and neurodevelopmental disorders. World Psychiatry.

[3] WHO (2022). ICD-11 Clinical Descriptions and Diagnostic Guidelines for Mental and Behavioural Disorders.

[4] Association A.P. (2013). Diagnostic and Statistical Manual of Mental Disorders.

[5] Bach B., First M.B. (2018). Application of the ICD-11 classification of personality disorders. BMC Psychiatry.

[6] Clark L.A., Nuzum H., Ro E. (2018). Manifestations of personality impairment severity: Comorbidity, course/prognosis, psychosocial dysfunction, and ‘borderline’ personality features. Curr. Opin. Psychol..

[7] Vanwoerden S., Stepp S.D. (2022). The Diagnostic and Statistical Manual of Mental Disorders, Fifth Edition, alternative model conceptualization of borderline personality disorder: A review of the evidence. Personal. Disord..

[8] Anderson J.L., Kelley S.E. (2022). Antisocial personality disorder and psychopathy: The AMPD in review. Personal. Disord..

[9] Samuel D.B., Balling C.E., Bucher M.A. (2022). The alternative model of personality disorder is inadequate for capturing obsessive-compulsive personality disorder. Personal. Disord..

[10] Miller J.D., Crowe M.L., Sharpe B.M. (2022). Narcissism and the DSM-5 alternative model of personality disorder. Personal. Disord..

[11] Hummelen B., Ulltveit-Moe Eikenaes I., Wilberg T. (2022). Is the alternative model for personality disorders able to capture avoidant personality disorder according to Section II of the DSM-5? A systematic review. Personal. Disord..

[12] Kwapil T.R., Barrantes-Vidal N. (2022). Schizotypal personality disorder in the alternative model for personality disorders. Personal. Disord..

[13] Bach B., Tracy M. (2021). Clinical Utility of the AMPD: A 10th Year Anniversary Review. Personal. Disord. Theory Res. Treat..

[14] Morey L.C., McCredie M.N., Bender D.S., Skodol A.E. (2022). Criterion A: Level of personality functioning in the alternative DSM-5 model for personality disorders. Personal. Disord..

[15] Widiger T.A., Hines A. (2022). The Diagnostic and Statistical Manual of Mental Disorders, Fifth Edition alternative model of personality disorder. Personal. Disord..

[16] Clark L.A., Watson D. (2022). The trait model of the DSM-5 alternative model of personality disorder (AMPD): A structural review. Personal. Disord..

[17] Bach B., Kramer U., Doering S., di Giacomo E., Hutsebaut J., Kaera A., De Panfilis C., Schmahl C., Swales M., Taubner S. (2022). The ICD-11 classification of personality disorders: A European perspective on challenges and opportunities. Bord. Personal. Disord. Emot. Dysregul..

[18] Bach B., Sellbom M., Skjernov M., Simonsen E. (2018). ICD-11 and DSM-5 personality trait domains capture categorical personality disorders: Finding a common ground. Aust. N. Z. J. Psychiatry.

[19] De Clercq B., De Fruyt F., Widiger T.A. (2009). Integrating a developmental perspective in dimensional models of personality disorders. Clin. Psychol. Rev..

[20] Sharp C., DeClercq B., Gratz K., Lejeuz C. (2020). Personality pathology in children and adolescents. Handbook of Personality Disorders.

[21] Shiner R.L., Allen T.A. (2013). Assessing personality disorders in adolescents Seven guiding principles. Clin. Psychol. Sci. Pract..

[22] Sharp C., Cano K., Bo S., Hutsebaut J., Huprich S.K. (2022). The assessement of personality function in adolescents. Ersonality Disorders and Pathology: Integrating Clinical Assessment and Practice in the DSM-5 and ICD-11 Era.

[23] Chanen A.M., Berk M., Thompson K. (2016). Integrating early intervention for borderline personality disorder and mood disorders. Harv. Rev. Psychiatry.

[24] Hutsebaut J., Videler A.C., Verheul R., Van Alphen S.P.J. (2019). Managing borderline personality disorder from a life course perspective: Clinical staging and health management. Personal. Disord.:Theory Res. Treat..

[25] Sharp C., Kerr S., Chanen A., Skodol A.E., Oldham J. (2021). Early identification and prevention of personality pathology: An AMPD informed model of clinical staging. The American Psychiatric Association Textbook of Personality Disorders.

[26] McAdams D.P. (2015). Three Lines of Personality Development A Conceptual Itinerary. Eur. Psychol..

[27] McAdams D.P. (2015). Tracing Three Lines of Personality Development. Res. Hum. Dev..

[28] McAdams D.P., Olson B.D. (2010). Personality Development: Continuity and Change Over the Life Course. Annu. Rev. Psychol..

[29] Sharp C., Vanwoerden S., Wall K. (2018). Adolescence as a Sensitive Period for the Development of Personality Disorder. Psychiatr. Clin. North Am..

[30] Sharp C., Oldham J. (2022). Nature and Assessment of Personality Pathology and Diagnosis. Am. J. Psychother..

[31] Tyrer P., Johnson T. (1996). Establishing the severity of personality disorder. Am. J. Psychiatry.

[32] Bender D.S., Morey L.C., Skodol A.E. (2011). Toward a model for assessing level of personality functioning in DSM-5, part I: A review of theory and methods. J. Personal. Assess..

[33] Morey L.C., Berghuis H., Bender D.S., Verheul R., Krueger R.F., Skodol A.E. (2011). Toward a Model for Assessing Level of Personality Functioning in DSM-5, Part II: Empirical Articulation of a Core Dimension of Personality Pathology. J. Personal. Assess..

[34] Tyrer P., Mulder R., Kim Y.R., Crawford M.J. (2019). The Development of the ICD-11 Classification of Personality Disorders: An Amalgam of Science, Pragmatism, and Politics. Annu. Rev. Clin. Psychol..

[35] Caspi A., Houts R.M., WBelsky D.W., Goldman-Mellor S.J., Harrington H., Israel S., Meier M.H., Ramrakha S., Shalev I., Poulton R. (2014). The p factor: One general psychopathology factor in the structure of psychiatric disorders?. Clin. Psychol. Sci..

[36] Clark L.A., Ro E. (2014). Three-Pronged Assessment and Diagnosis of Personality Disorder and Its Consequences: Personality Functioning, Pathological Traits, and Psychosocial Disability. Personal. Disord.-Theory Res. Treat..

[37] Shields A.N., Giljen M., Espana R.A., Tackett J.L. (2021). The p factor and dimensional structural models of youth personality pathology and psychopathology. Curr. Opin. Psychol..

[38] Wright A.G.C., Ringwald W.R. (2022). Personality disorders are dead; long live the interpersonal disorders: Comment on Widiger and Hines (2022). Personal. Disord..

[39] Widiger T.A., Bach B., Chmielewski M., Clark L.A., DeYoung C., Hopwood C.J., Kotov R., Krueger R.F., Miller J.D., Morey L.C. (2019). Criterion A of the AMPD in HiTOP. J. Pers. Assess..

[40] Busmann M., Wrege J., Meyer A.H., Ritzler F., Schmidlin M., Lang U.E., Gaab J., Walter M., Euler S. (2019). Alternative Model of Personality Disorders (DSM-5) Predicts Dropout in Inpatient Psychotherapy for Patients With Personality Disorder. Front. Psychol..

[41] Sharp C., Kerr S., Barkauskiene R. (2022). The incremental utility of maladaptive self and identity functioning over general functioning for borderline personality disorder features in adolescents. Personal. Disord..

[42] Goth K., Birkhölzer M., Schmeck K. (2018). LoPF-Q 12–18 (Levels of Personality Functioning Questionnaire) German Version: A Self-Report Questionnaire for Measuring Personality Functioning in Adolescence-Short Description. http://www.academic-tests.com.

[43] Sharp C., Vanwoerden S. (2018). Culture-Adapted Version English USA of the Self-Report Questionnaire LoPF-Q 12-18 (Levels of Personality Functioning Questionnaire; authors Goth & Schmeck)—Short Manual. Academic-Tests. 2018. (Unpublished translation). Acad.-Tests.

[44] Cosgun S., Goth K., Cakiroglu S. (2021). Levels of Personality Functioning Questionnaire (LoPF-Q) 12–18 Turkish version: Reliability, validity, factor structure and relationship with comorbid psychopathology in a Turkish adolescent sample. J. Psychopathol. Behav. Assess..

[45] Goth K., Birkholzer M., Schmeck K. (2018). Assessment of personality functioning in adolescents with the LoPF-Q 12-18 Self-Report Questionnaire. J. Personal. Assess..

[46] Kerr S., McLaren V., Cano K., Vanwoerden S., Goth K., Sharp C. (2022). Levels of Personality Functioning Questionnaire 12–18 (LoPF-Q 12–18): Factor structure, validity, and clinical cut-offs. Assessment.

[47] Goth K., Schmeck K. (2018). AIDA (Assessment of Identity Development in Adolescence) German Version: A Self-Report Questionnaire for Measuring Identity Development in Adolescence-Short Manual. https://www.academic-tests.com.

[48] Sharp C., Vanwoerden S., Odom A., Foelsch P. (2018). Culture-adapted version English USA of the Self-Report Questionnaire AIDA (Assessment of Identity Development in Adolescence; authors Goth & Schmeck)- Short manual. Offenb. Acad.-Tests.

[49] Goth K., Foelsch P., Schlüter-Müller S., Birkhölzer M., Jung E., Pick O., Schmeck K. (2012). Assessment of identity development and identity diffusion in adolescence- Theoretical basis and psychometric properties of the self-report questionnaire AIDA. Child Adolesc. Psychiatry Ment. Health.

[50] Kassin M., De Castro F., Arango I., Goth K. (2013). Psychometric properties of a culture-adapted Spanish version of AIDA (Assessment of Identity Development in Adolescence) in Mexico. Child Adolesc. Psychiatry Ment. Health.

[51] Sharp C., McLaren V., Musetti A., Vanwoerden S., Hernandez Ortiz J., Schmeck K., Birkhoelzer M., Goth K. (2022). The Assessment of Identity Development in Adolescence (AIDA) questionnaire: First psychometric evaluation in two North American samples of young people. J. Personal. Assess..

[52] Achenbach T.M., McConaughy S.H., Ivanova M.Y., Rescorla L.A. (2011). Manual for the ASEBA brief problem monitor (BPM). https://documents.acer.org/ASEBA_Brief_Problem_Monitor_Manual.pdf.

[53] Achenbach T.M. (2009). The Achenbach System of Empirically Based Assessment (ASEBA): Development, Findings, Theory, and Applications.

[54] Richter J. (2015). Preliminary evidence for good psychometric properties of the Norwegian version of the Brief Problems Monitor (BPM). Nord. J. Psychiatry.

[55] Krueger R.F., Derringer J., Markon K.E., Watson D., Skodol A.E. (2013). The Personality Inventory for DSM–5, Brief Form (PID-5-BF)-Adult.

[56] Krueger R.F., Eaton N.R., Clark L.E., Watson D., Markon K.E., Derringer J., Skodol A., Livesley W.J. (2012). Deriving an empirical structure of personality pathology for DSM-5. J. Personal. Disord..

[57] Fossati A., Somma A., Borroni S., Markon K.E., Krueger R.F. (2017). The Personality Inventory for DSM-5 Brief Form: Evidence for reliability and construct validity in a sample of community-dwelling Italian adolescents. Assessment.

[58] Romero Triñanes E., Alonso Vilar C. (2019). Maladaptative personality traits in adolescence: Behavioural, emotional and motivational correlates of the PID-5-BF scales. Psicothema.

[59] Korycinski K.M. (2017). A Psychometric Evaluation of the Brief Form of the PID-5 in an Inpatient Adolescent Sample. Master’s Thesis.

[60] Sharp C., Steinberg L., Temple J., Newlin E. (2014). An 11-item measure to assess borderline traits in adolescents: Refinement of the BPFSC using IRT. Personal. Disord. Theory Res. Treat..

[61] Crick N.R., Murray-Close D., Woods K. (2005). Borderline personality features in childhood: A short-term longitudinal study. Dev. Psychopathol..

[62] IBM Corp (2022). IBM SPSS Statistics for Windows.

[63] Garcia D.J., Skadberg R.M., Schmidt M., Bierma S., Shorter R.L., Waugh M.H. (2018). It’s not that difficult: An interrater reliability study of the DSM-5 Section III Alternative Model for Personality Disorders. J. Personal. Assess..

[64] Sharp C. (2022). New data toward fulfilling the promise of the ICD-11 severity criterion. Personal. Ment. Health.

